# Is the Schwabe Organ a Retained Larval Eye? Anatomical and Behavioural Studies of a Novel Sense Organ in Adult *Leptochiton asellus* (Mollusca, Polyplacophora) Indicate Links to Larval Photoreceptors

**DOI:** 10.1371/journal.pone.0137119

**Published:** 2015-09-14

**Authors:** Lauren H. Sumner-Rooney, Julia D. Sigwart

**Affiliations:** 1 School of Biological Sciences, Queen’s University Belfast, Belfast, Northern Ireland; 2 Queen’s Marine Laboratory, Queen’s University Belfast, Portaferry, Northern Ireland; United States Department of Agriculture, Beltsville Agricultural Research Center, UNITED STATES

## Abstract

The discovery of a sensory organ, the Schwabe organ, was recently reported as a unifying feature of chitons in the order Lepidopleurida. It is a patch of pigmented tissue located on the roof of the pallial cavity, beneath the velum on either side of the mouth. The epithelium is densely innervated and contains two types of potential sensory cells. As the function of the Schwabe organ remains unknown, we have taken a cross-disciplinary approach, using anatomical, histological and behavioural techniques to understand it. In general, the pigmentation that characterises this sensory structure gradually fades after death; however, one particular concentrated pigment dot persists. This dot is positionally homologous to the larval eye in chiton trochophores, found in the same neuroanatomical location, and furthermore the metamorphic migration of the larval eye is ventral in species known to possess Schwabe organs. Here we report the presence of a discrete subsurface epithelial structure in the region of the Schwabe organ in *Leptochiton asellus* that histologically resembles the chiton larval eye. Behavioural experiments demonstrate that *Leptochiton asellus* with intact Schwabe organs actively avoid an upwelling light source, while *Leptochiton asellus* with surgically ablated Schwabe organs and a control species lacking the organ (members of the other extant order, Chitonida) do not (Kruskal-Wallis, H = 24.82, df = 3, p < 0.0001). We propose that the Schwabe organ represents the adult expression of the chiton larval eye, being retained and elaborated in adult lepidopleurans.

## Introduction

**Fig 1:**
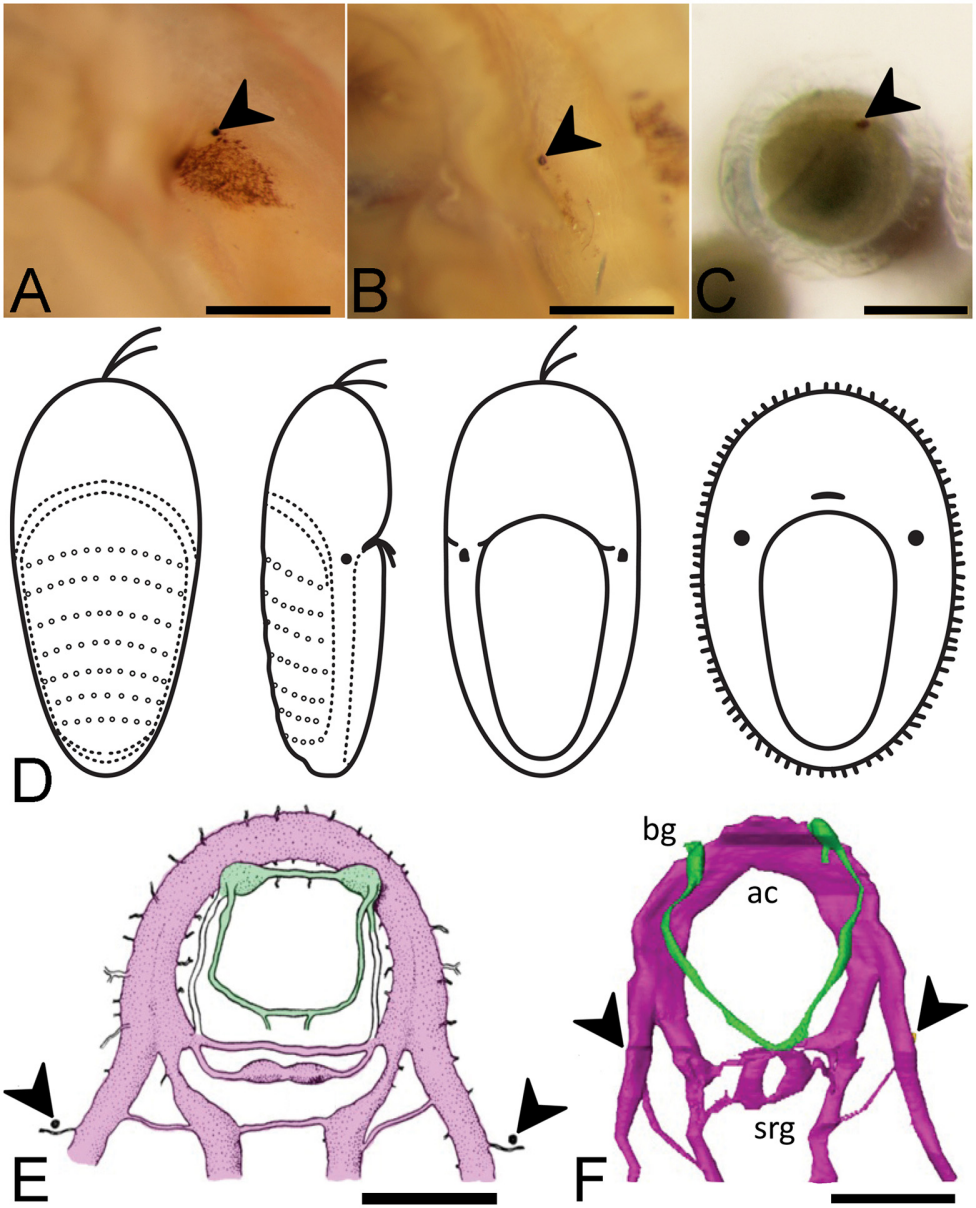
The Schwabe organ in *Leptochiton asellus* and the larval eye. A, Schwabe organ in a fresh glutaraldehyde-fixed specimen. B, Several months after death. Note the persistence of a concentrated dot at the anterior edge (chevron). Scale bar, 500 μm. C, The larval eye in *Lepidochitona cinerea in ovo* (chevron). Scale bar, 200 μm. D, The ventral migration of the larval eye of *Leptochiton asellus*. Left to right; dorsal view of developing trochophore with visible shell fields; lateral view with larval eye visible; ventral view with larval eyes having migrated to position lateral to the tip of the foot; metamorphosed larva with larval eyes in final position, reminiscent of the Schwabe organ. Adapted from Christiansen, 1954. E, The position of the larval eye in the anterior nervous system of *Lepidozona mertensii* (purple, adapted from Heath, 1904). Buccal ganglia and nerves are highlighted in green. Scale bar, 1 mm. F, The position of the Schwabe organ in the anterior nervous system of adult *Leptochiton asellus*. Scale bar, 1 mm. ac, anterior commissure; bg, buccal ganglia (in green); srg, subradular ganglia.

Chitons (Mollusca, Polyplacophora) have a flattened, ovoid body with a muscular foot protected by eight dorsal shell valves. The chiton nervous system is considered to represent the plesiomorphic condition amongst molluscs, lacking discrete ganglia and with few advanced sensory organs [1,2]. A previously unknown sensory organ, the Schwabe organ, has recently been discovered and described throughout one of the two taxonomic orders of chitons [3]. This is as a stripe of brownish pigmentation, visible to the naked eye, which extends posteriorly and laterally from either side of the mouth towards the top of the foot (Fig 1A and 1B). The pigmented epithelium is innervated by bundles of nerve fibres and contains multiciliary cells and round basal cells with epithelial projections, both of which could be sensory [3]. However, no specialised structures are visible to surface examination by SEM, and the function of the Schwabe organ remains unknown.

Though chitons do not possess cephalic eyes, all members of the class (both taxonomic orders) have specialised pores in their dorsal shells, the aesthetes [4]. In many species, the aesthetes contain photoreceptors and mediate negative light response behaviour, though the function has never been documented for members of Lepidopleurida [4–9].

Larval development has been described for relatively few species of chitons, including only two species in the order Lepidopleurida: *Leptochiton asellus* [10] and *Lepidopleurus cajetanus* [11]. For all species where early ontogeny has been described, chiton trochophore larvae possess larval eyes (Fig 1C). These are post-trochal pigmented photosensitive organs that are thought to contribute to the settlement behaviour of larvae, as several species’ larvae are negatively phototactic [12]. Chitons undergo metamorphosis from trochophore to adult form during settlement, forming a ventral foot and dorsal shell fields. Several authors have described the anatomical migration of the apical larval eyes to the dorsal side during metamorphosis, before they become covered by the growing shell field [reviewed in 12]. Heath [13] noted the persistence of the larval eye pigment and suggested it remained a functional eye for as long as it was visible at the dorsal side in developing and early post-metamorphic animals. The eye is ventrally visible for some time in species in Chitonida [14], and Kowalevsky [15] described the eye sinking beneath the epithelium and persisting to adulthood in *Chiton polii*. However, clear ventral migration of the larval eye has only been reported in *Leptochiton asellus* [10]; Naef’s drawings of *Lepidopleurus cajetanus* larvae are ambiguous in this respect [11]. This ventral migration in Lepidopleurida results in the larval eye attaining a position reminiscent of the Schwabe organ in adults (Fig 1D). In fact this similarity is not just superficial, but is also reflected in the neuroanatomy. The position of the Schwabe organ is ventral to the lateral nerve cord, and slightly lateral and posterior to the origin of the first lateropedal connective [3; Fig 1F], corresponding precisely to the location of the larval eye [13; Fig 1E].

Sigwart et al. [3] described a bleaching effect that occurs in the pigmented region of the Schwabe organ and speculated this bleaching explained why the sense organ had not been described historically. Further observations of fixed specimens of *Leptochiton asellus* show that even when most of the pigment has faded, there is a small dot of dense pigment that persists (Fig 1A and 1B). In *Leptochiton asellus* this dot is found anterior to the centre of the Schwabe organ sunken beneath the surface, and in live animals appears to contain more concentrated pigmentation, suggesting it is an important component of the Schwabe organ.

Determining the function of a sensory structure such as the Schwabe organ presents numerous challenges that can be managed in a number of ways; structurally (using anatomical or developmental observations) or functionally (directly demonstrating the use of the Schwabe organ through behavioural responses). Here, we combine these techniques in order to further explore the possibility of a link between the Schwabe organ and the larval eye.

## Materials and Methods

### Specimens

Specimens of *Leptochiton asellus* (Lepidopleurida) and *Lepidochitona cinerea* (Chitonida) were collected intertidally from Strangford Lough, Northern Ireland, and housed in aquaria supplied with filtered seawater at 8–10°C. Additional specimens of *Leptochiton asellus* were collected by dredging in Kristineberg, Sweden, and were housed in standing-water aquaria maintained at 8–10°C and supplied with filtered seawater. Field collections were undertaken with appropriate permissions via the Queen's University Belfast Marine Laboratory (Strangford Lough, N. Ireland) and the Sven Lovén Centre for Marine Sciences (Gullmar fjord, Sweden). Therefore no specific individual permissions were required for these locations and activities.

### Histology

Specimens of *Leptochiton asellus* were fixed in 4% glutaraldehyde in cacodylate buffer (pH 7.2) for 48 hours. Tissue blocks containing the Schwabe organ were excised, washed in dilute cacodylate buffer and post-fixed in 1% osmium tetroxide in cacodylate buffer for two hours. Samples were washed in dilute cacodylate buffer and dehydrated in an acetone series according to Ruthensteiner [16]. Dehydrated samples were kept in a 1:1 mixture of 100% acetone and epoxy resin overnight, allowing the acetone to evaporate, and embedded in Epon epoxy resin according to the manufacturer’s instructions (Agar Scientific).

Serial semi-thin sections (1.5 μm) were taken using a diamond knife (HistoJumbo 8mm 45°, DiATOME, Switzerland) on an automated microtome (Leica RM2255). Slides were stained with Richardson’s solution [17] and visualised on an Olympus SX17 light microscope.

Ultra-thin sections (60–80 nm) were taken on an automated Ultracut E ultramicrotome (Reichert) using a diamond knife (DiATOME) and collected on copper grids. Sections were stained with uranyl acetate and lead citrate and visualised on a Philips CM100 transmission electron microscope at 60 kV.

### Behavioural experiments

In order to test potential photosensitivity of the Schwabe organ, subjects were monitored for responses to upwelling light (i.e. illumination from below the surface the animals were crawling on). Healthy specimens of *Leptochiton asellus* (n = 40, 6–15 mm) and *Lepidochitona cinerea* (n = 40, 5–13 mm) were selected for the study. Twenty animals of each species were selected at random for ablation treatment. They were relaxed in a 7% MgCl_2_ solution for twenty minutes before ablations were performed on the Schwabe organ in *Leptochiton asellus* and the corresponding epithelial tissue either side of the mouth in *Lepidochitona cinerea* using a fine 125 mm extended-tip high temperature cautery pen (FIAB, Florence). Following surgery, specimens were given three days for recovery before experiments. During this period, all subjects (ablated and intact) were kept in starvation conditions in clean holding aquaria.

**Fig 2:**
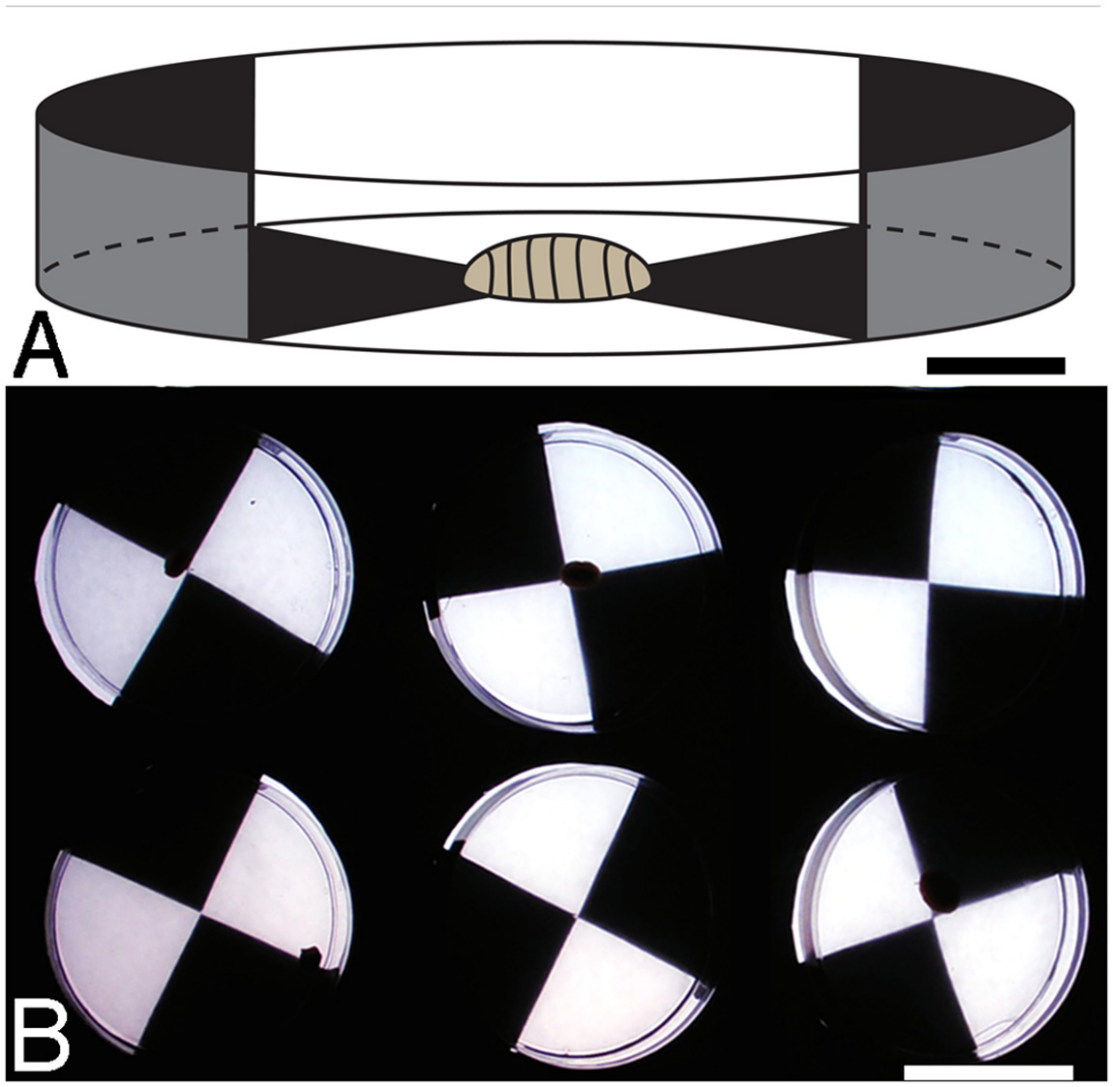
Light response experiments. A, Chitons were placed in circular arenas with two blackened and two lit quadrants of equal size. Scale bar, 1 cm. B, Arenas were placed on a horizontal lightbox, illuminating the lit quarters from below. Scale bar, 5 cm.

A light box (DW Viewboxes, Milton Keynes) was adapted using adhesive black plastic film to create 20 individual arenas. Circular arenas (diameter 87 mm) were divided into four quarters: two blacked out and two covered by a wax paper diffuser, allowing white light from the light box to penetrate. Arena orientation was randomised to eliminate the effects of other potential stimuli and sterile petri dishes were filled with chilled (10°C) filtered seawater and placed over each arena (Fig 2). A 5 mm Perspex sheet was placed between the light box and the dishes to diffuse any heat. Each subject was placed at the centre of an arena at a randomised orientation and allowed 10 minutes to uncurl in complete darkness before trials began. The position of each chiton was digitally recorded at five minute intervals over a four hour trial period, during which time they were not disturbed. This temporal resolution was found to be sufficient to reliably indicate their positions within the arena. Experiments took place in an otherwise totally darkened room under stable temperature conditions and in the absence of any other stimuli.

#### Analysis

Time lapses were analysed blind. Position data were extracted from the images in ImageJ (National Institutes of Health). From these, the relative percentages of time spent on light and dark surfaces, subjects’ initial choices, and whether they settled on one or the other for an hour or more were all recorded. Subjects were scored as present in a light section if any part of their body was visible on a lit patch. Chitons were considered to have “settled” if they remained on either dark (not visible to observer) or light (visible) areas for one hour or more (12 images). All statistical analyses were performed using R, Kruskal-Wallis post hoc analyses were performed using the pgirmess package [18,19].

## Results

### Histology

**Fig 3:**
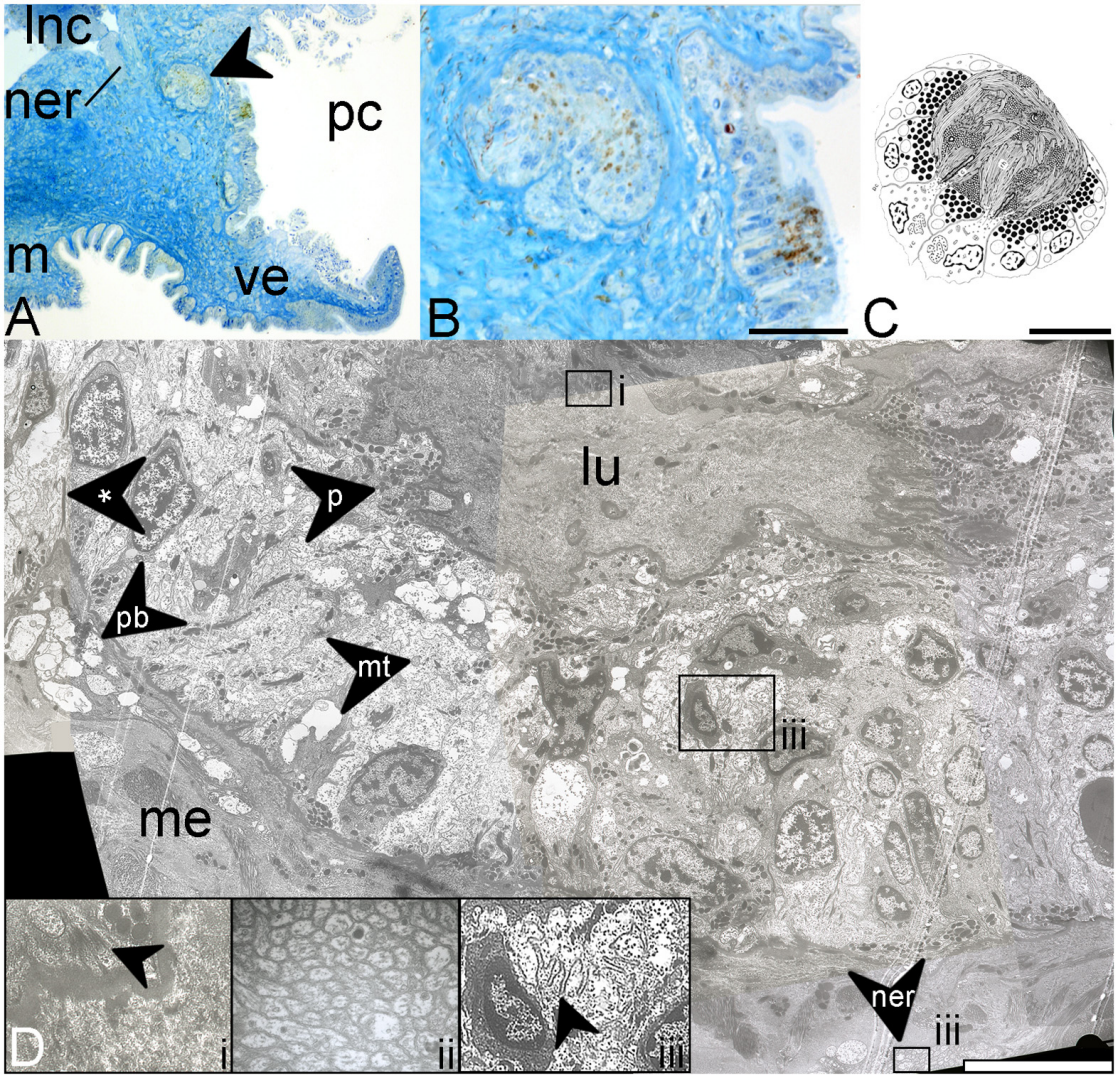
Anatomy and ultrastructure of the adult larval eye within the chiton Schwabe organ. A, Semi-thin section showing pigmented epithelium and secondary pigmented structure (chevron) in the Schwabe organ in *Leptochiton asellus*; the pallial cavity is to the right as shown. lnc, lateral nerve cord; m, mouth; ner, nerve. Scale bar, 100 μm. B, Semi-thin section showing epithelial and secondary pigmented structures observed the region of the Schwabe organ. Scale bar, 50 μm. C, Ultrastructure of the larval eye (from Fischer, 1980). Scale bar, 2 μm. D, Ultrastructure of the pigmented subepithelial structure. Insets from main image; i, putative ciliary roots (chevron); ii, nerve bundles; iii, membrane folding (chevron). lu, lumen; me, mesoderm; mt, mitochondrion; ner, nerve; p, pigment; pb, large pale bodies; asterisk, elongated dark structure. Scale bar, 10 μm.

Histological and ultrastructural investigation of the Schwabe organ revealed striking similarities to the larval eye as described by Fischer [20]. At the anteriormost end of the Schwabe organ pigment patch is a raised patch of pigmented epithelium around 70 μm across (Fig 3A and 3B). It contains two to three pseudostratified layers of long slender cells, around 50 μm high. Many of these cells, particularly at the centre of this raised patch, contained brownish pigment at their apical side (Fig 3B). Several also appeared to bear cilia, but these could not be definitively identified in TEM images.

A large structure was present directly beneath the raised epithelium, ventral to the lateral nerve cord. This is ovoid, pigmented, and around 100 μm across (Fig 3A and 3B). Given its size and position we believe that this represents the sunken concentrated pigment dot observed at the anterior side of the Schwabe organ. It is lined by cells resembling epithelial cells, which appear to be stacked or pseudostratified in places and are separated from the rest of the mesoderm by a basal lamina (Fig 3D). Some cells are columnar and have large, ovoid nuclei (around 5 μm high) and others are smaller interdigitating cells with smaller, rounded nuclei (around 2.5 μm across). The latter tend to be basally located. Most cells have a pale but grainy cytoplasm, and vesicles of several types are present throughout. Dark, elongate structures are also present within several cells and appear to project towards the apical side (Fig 3D). Pigment granules (up to 0.7 μm across) are visible both apically and basally in many of these cells, but with apical concentrations generally being much higher. Pigmented cells tend to have slightly darker cytoplasm. Non-pigmented cells contain many mitochondria and have a paler cytoplasm. Membrane folding is often visible at the apical sides of these cells. From semi-thin sections we can identify a large nerve projecting from the lateral nerve cord towards this structure, and nerve fibres are present beneath the basal lamina and occasionally within the basal parts of the epithelial layer. At one side, several large pale bodies (resembling similar structures within the epithelium) and nerve fibres are present beneath the basal lamina (Fig 3D). At the apical edge of this cell layer (the interior or lumen of the overall rounded structure) is a second lamina which surrounds a central body. This appears to be fairly homogenous in composition and stains darkly. There are many membranous and occasional round structures present (roughly 0.3 μm across) which may be microvillous. It also contains elements resembling the cilia Fischer considered to form rhabdoms in the larval eye of *Lepidochitona cinerea*, with putative ciliary roots visible at the apical side of several cells [20; Fig 3C and 3D].

### Behavioural experiments

**Fig 4:**
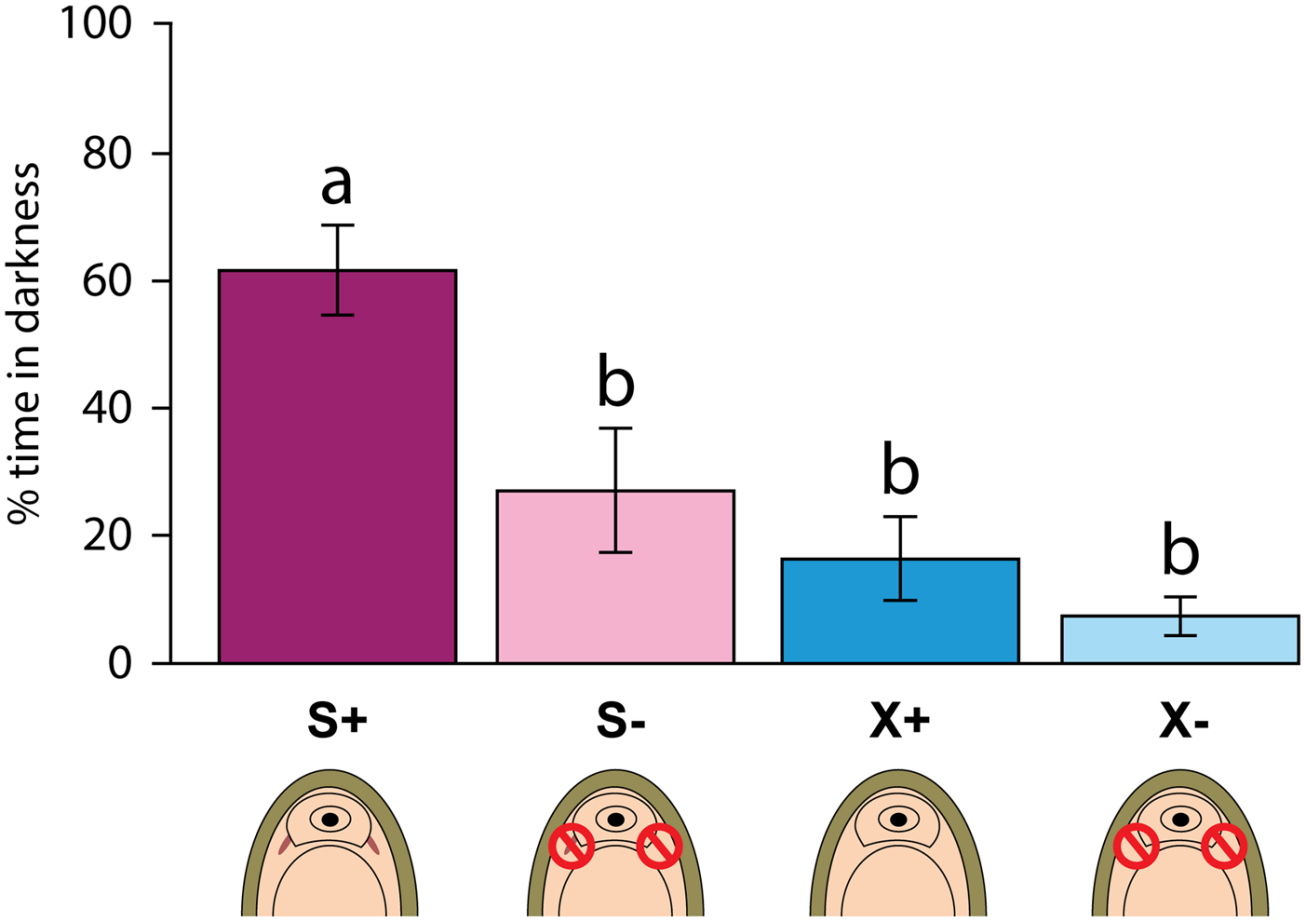
Behavioural experiments to test response of the Schwabe organ to upwelling light. Percentage of time spent in the darkened quadrants by the four subject groups. S indicates innate presence of Schwabe organ (*Leptochiton asellus*), X indicates innate absence of Schwabe organ (*Lepidochitona cinerea*); + indicates subject intact,–indicates ablation surgery. Error bars indicate standard error and are intended as visual guidelines only. Letter codes above bars denote statistically equivalent time spent in the darkened quadrants between treatment groups (based on KW post hoc pairwise comparisons).

Behavioural experiments indicated that subjects with intact Schwabe organs were significantly more likely than other chitons to avoid areas of upwelling light (Fig 4). The proportion of time spent in darkened areas differed significantly between the four treatment groups (Kruskal-Wallis, H = 24.82, df = 3, p < 0.0001). The intact *Leptochiton asellus* spent significantly more time in the dark areas than the ablated *Leptochiton asellus* (KW pairwise comparisons, H = 21.64, df = 1, p < 0.05) and both groups (ablated and intact) of *Lepidochitona cinerea* (KW pairwise comparisons, ablated *Lepidochitona cinerea* H = 29.88, df = 1, p < 0.005; intact *Lepidochitona cinerea* H = 23.67, df = 1, p < 0.005). The likelihood of a chiton settling (as opposed to moving continuously) was not affected by treatment (χ^2^ = 1.862, df = 3, p > 0.05); however, intact *Leptochiton asellus* were more likely to settle in a dark space than any other subjects (χ^2^ = 24.169, df = 3, p < 0.001).

## Discussion

From the evidence presented here, we conclude that the Schwabe organ is paedomorphic, an elaboration of the larval eye which persists to adulthood in *Leptochiton asellus*. The positional homology of the larval eye and the ‘dot’ element of the Schwabe organ indicate that the larval and adult structures are likely to be closely linked, and they share several ultrastructural features. Further, the results of behavioural experiments demonstrate that the Schwabe organ has a photoreceptive function, which could persist in a retained larval eye.

Although there are no data available on the ultrastructure of the larval eye in *Leptochiton asellus* trochophores, or other species in Lepidopleurida, the structure observed beneath the epithelial surface of *Leptochiton asellus* strongly resembles available descriptions of the chiton larval eye in a species in Chitonida [20,21]. This structure, which appears to be an embedded larval eye in adult *Leptochiton asellus*, is considerably larger than the larval eye in trochophores of *Lepidochitona cinerea* or *Katharina tunicata* (around 100 μm across rather than 15 μm or 25 μm respectively) [20,21], but this could be interspecific and/or ontogenetic difference. Its overall structure is very similar, being a discrete rounded capsule of epithelial-type cells which appears to be innervated by the lateral nerve cord. Tall pigmented cells are interspersed with non-pigmented (perhaps sensory) cells containing many mitochondria and vesicular bodies, and these surround a central lumen which may contain cilia and microvilli [20,21]. Both are also associated with nerve bundles at their basal sides.

The possibility of a fundamental link between the Schwabe organ, an adult sensory system, and the larval eye, has interesting developmental implications. Voronezhskaya et al. [22] found that none of the neurons in the larval central nervous system persisted to adulthood in *Ischnochiton hakodadensis*, a species in Chitonida that does not possess a Schwabe organ. This may reflect a fundamental difference between the two orders of chitons, and that ventral migration and persistence of functional larval eyes is tied directly to the Schwabe organ as a feature found only in Lepidopleurida. Whether this is a novelty exclusive to Lepidopleurida or a plesiomorphic character that has been lost in Chitonida is difficult to determine, despite the expansion and elaboration of the larval eye apparent in the Schwabe organ. Beyond polyplacophorans, there is evidence of not only the survival of sensory neurons through development but an entire gustatory larval sense organ has been found to persist and become elaborated during metamorphosis in *Drosophila melanogaster* [23–26]. It is therefore certainly possible that the larval eye might survive, or even expand, through metamorphosis despite significant reorganisation of both the nervous system and the body as a whole.

Based on our behavioural experiments, the Schwabe organ is apparently photosensitive and mediates light avoidance behaviour. Among our experimental trials, multiple subjects of intact *Leptochiton asellus* approached the edge of a darkened quadrant head-on, before pausing and turning back. Many in this group also passed between the two darkened quadrants directly over the centre of the arena, maintaining minimal light exposure. These behaviours were not observed in *Lepidochitona cinerea* and rarely in ablated *Leptochiton asellus*. Specimens of *Leptochiton asellus* with an intact Schwabe organ differed markedly more from the two groups of *Lepidochitona cinerea* than they did from ablated conspecifics (Fig 4). This could be a result of variable efficacy of the ablation treatment, which may not have rendered all Schwabe organs completely dysfunctional. The light-dark scoring system was designed to give conservative estimates; chitons with any visible parts were considered to be in light sections, as the basic assumption is that they will behave in a photonegative way. This led to some chitons that were for the most part in dark sections being scored as in light sections, if any small part of them was visible, and so light-indifferent animals appear to spend an increased amount of time in the lit areas. Despite the subsequent inflation of light versus dark scores, it is still clear that intact *Leptochiton asellus* avoided light exposure as much as possible.

Negative phototaxis (in response to downcast light) has been documented in many species of chitons and presumably contributes to their staying hidden from predators beneath rocks [27,28]. Where photosensitive aesthetes are present, these are likely to play a role in facilitating this behaviour [4,5]. Both *Lepidochitona cinerea* and *Leptochiton asellus* possess dorsal aesthetes, which were not affected in our experimental treatments. Although the function of aesthetes in *Leptochiton asellus* has not been directly documented, *Lepidochitona cinerea* is among the many species shown to respond to downcast light [5]. Thus, the absence of avoidance behaviour in *Lepidochitona cinerea* and ablated *Leptochiton asellus* provides strong evidence that the response observed in *Leptochiton asellus* is not attributable to aesthete activity and that the upwelling-only nature of the light source was effective.

It is clear from our results that the Schwabe organ represents a photosensitive adult expression of the larval eye, but this is not an entirely satisfactory explanation to the role of the sense organ in the natural history of these animals. The Schwabe organ is present in all genera in the chiton order Lepidopleurida [3], yet the majority of species in this clade inhabit the deep sea [29,30]. Many of the deep sea lineages have extensive and elaborate pigment patches; for example the genus *Nierstraszella* lives at depths from 200–1760 m yet possesses one of the largest Schwabe organs yet observed [31]. Furthermore the Schwabe organ is positioned on the ventral surface of the body. Thus, it is on the under-side of animals that live attached to rocks, often in depths below the penetration of solar light. Of course, the existence of a phototactic response in the intertidal species *Leptochiton asellus* does not guarantee the equivalent response in all of the approximately 120 deep-sea species in Lepidopleurida. Equally puzzling is the absence of the Schwabe organ in the other living order of chitons, Chitonida. These usually shallow-water species might speculatively have more biological gain from a photosensitive sensory organ, and it is exclusively in this order that the advanced shell eyes are present. Given that specimens exhibited negative phototaxis it seems unlikely that the Schwabe organ could be used for any kind of feeding activity (on bioluminescent biofilms, for example). An aversion to bioluminescent or phosphorescent surfaces could help avoid detection by visual predators in an otherwise darkened environment, but this seems an unlikely explanation and the Schwabe organ has possibly become more of an enigma than before.

This is the first description of a paired, ‘eye-like’ structure in adult chitons. Photoreception in chitons was previously considered to be restricted to the aesthetes and specialised shell eyes, but we have demonstrated that the Schwabe organ represents another photosensitive structure which is likely to be present throughout an entire taxonomic order.
